# A Case of Suppressed Gonorrhœa

**Published:** 1888-06

**Authors:** E. W. Berridge

**Affiliations:** London


### A CASE OF SUPPRESSED GONORRHŒA.

E. W. Berridge, M. D., London.

April 16th, 1887, I was consulted by a colleague about one of his patients, a man about thirty or thirty-five years of age. The history of the case is as follows: Twenty-four days ago gonorrhœa appeared, after an impure coitus five or six days previously. It was his first attack. His physician treated him with low potencies, which relieved the acute symptoms. On April 8th he got chilled during a walk without an overcoat, the weather being cold; this suppressed the discharge and caused swelling and pain in the left testis. On April 15th, as his bowels had not acted for three days, his physician gave him a dose of Castor oil. This operated several times, but caused him to again catch cold when at the closet. Since eight A. M. this morning he has been in dreadful pain. His physician gave him a low potency of *Belladonna*, which was "like so much water on a duck's back." So in the afternoon he asked me to see the patient with him. I saw him between seven and eight P. M., and found him in the following condition: Since eight A. M. paroxysms of most intense pain, which have increased till the present time. The paroxysms come on about every ten minutes; the pain is most intense, making him writhe, groan, and weep, and grasp the bedposts tightly. He is a strong man, who knows what severe pain is and can bear it well, but he says he never felt anything like this. The pain begins in left iliac region and extends to left groin and half-way down front of left thigh. It is *as if a fluid were forcing its way there*, something like an injection, and it ends in a sudden pain in anterior part of middle of left thigh, as from a jagged knife. Between the paroxysms there is throbbing in different parts of left groin and front of left thigh; left inguinal gland enlarged and tender; left testis swollen; scarcely any discharge. Before the paroxysms come on he had a dull pain, extending from left iliac region to left mid-thigh, with frequent digs in various parts thereof. This pain he has still, but it is nothing compared to the paroxysms; tongue white; pulse quick.

### Diagnosis of the Remedy.

Taking the characteristic pain of the paroxysms, three medicines were indicated for study—*Coccus cacti*, *Glonoine*, and *Opium*.

Opium has "sensation as if some liquid were moving up and down in left thigh."

*Glonoine* has "gradually increasing pressure from forehead toward vertex, as if a liquid were being pressed upward from root of nose and forced at the back through *sinus longitudinalis* with constantly increasing force. This pressure grows so severe that a general perspiration breaks out, with redness of the face and great anxiety."

*Coccus cacti* has "violent, raging pain, extending from right eye along squamous portion of temporal bone on its inner side to occiput. It seems as though a fluid were injected paroxysmally into a small blood vessel."

Of these three remedies Opium was rejected, as the symptom is evidently a painless sensation. The *Glonoine* symptom does not show the paroxysmal character of the pain, and its concomitants were absent in the patient. *Coccus cacti* has this feature in a marked degree, and, moreover, has produced "burrowing tensive, dragging, and drawing pain in hypochondriac, pubic, and inguinal regions."

I dissolved a few pellets of *Coccus cacti*$^{cm}$ (Fincke) in a glass of water, gave him a spoonful, and told him to take a spoonful every hour till decidedly better, then every two hours. The first dose was taken about seven P. M.

April 17th, eleven A. M.—Says the action of the medicine was wonderful, though when he saw me dissolve the pellets he felt that it could do no good, and that he must have Chloroform. Allopathic physicians had always told him that in severe pain nothing but Morphine or anæsthetics can relieve promptly (some pseudo-homœopaths say so too). He now tells me that after the first dose the paroxysms continued to increase in severity and frequency for about an hour, then for the next hour after the second dose at longer intervals of about fifteen minutes. About nine P. M., after third dose, the paroxysms ceased, and did not return, except about fifteen minutes past two A. M.; has only slept from three A. M. to seven A. M.; scarcely any discharge; has taken the medicine every hour when awake; once he waited one hour and a half, but the constant pain (not the paroxysms) began to increase, and he also felt it worse after the four hours sleep, apparently from want of the medicine. He has now occasional acute pain in groin and dull pain in thigh; inguinal gland less tender; tongue less white; pulse slower; he feels altogether much better and is astonished at the result. So was also his former physician, and his countenance, when I told him the potency given, was an interesting study. He paid me the high compliment of asking me to take the case into my own hands, which I did, and continued the medicine every three hours.

April 18th, nine A. M.—Says that last night and the night before, as soon as he turned on to his left side the wind seemed to collect in a ball in left groin and roll over to right abdomen, and then pass away by the bowels. Yesterday he felt free from severe pain till quarter to seven P. M.; felt generally better in the morning, but had a few twinges of pain in the afternoon. Since quarter to seven P. M. has had the constant pain with a varying degree of acuteness, but no paroxysms except one severe one at quarter to seven P. M. and two more lesser ones before eight P. M. Has had no return of the "fluid" pain, except a little in the first of these three paroxysms. Has had snatches of sleep during night, discharge returning. The pain is now the same dull pain from left iliac region to mid-thigh, with frequent digs in various spots thereof, just as it was before the paroxysms came on, only less severe; he has had this since quarter to seven P. M. He took the medicine every two or three hours till quarter to seven P. M., and since then every hour while awake. As an aggravation of the symptoms seemed to have commenced I stopped the medicine.

April 19th, nine A. M.—Pain remained about the same yesterday morning till after one P. M.; then the excitement of some legal business increased the pains till about six P. M., when he became easier again. Had a good night, sleeping about seven hours. To-day he feels wonderfully easier—"happiness itself;" can hardly believe it. This morning he can stretch the leg out, which he could not before. Rather more discharge. Left testis still swollen.

April 20th, nine P. M.—Yesterday the excitement of legal business again brought on the dull pain from six P. M. to four A. M., but to-day it has not troubled him much. Testis still swelled. Discharge considerably increased. Has had no stool for six days and has a cough, just as he had the last time he was constipated. I gave him an enema of warm water, which acted very profusely in about fifteen minutes.

April 21st, nine A. M.—He had a fair night, with very little pain. Discharge ceased. Testis smaller, and no longer tender. Still pain in groin and thigh, but much less than yesterday.

April 23d.—Has slept very well. Only a little pain for the last two days at the end of urination. Contractive pain all around lower abdomen, groins, and upper thighs, all around in a circle. Scarcely any discharge. Testis smaller and without tenderness.

April 26th.—Reports that pain has not returned since the morning of 24th, except a very little while walking. Slight discharge. Scarcely any swelling of testis. Sleeps well.

May 6th.—Writes to say that he is quite well, and has been at work in his office for the last four days.

COMMENTS.—1. The peculiar "fluid" pain occurred in the patient in the abdominal and femoral regions, but was cured by a remedy producing a similar pain in the head. When a medicine possesses the property of producing a very peculiar sensation in one part, it will often cure it when occurring in other parts, as I have several times verified. This shows the value of Hering's *Guiding Symptoms*, where a special rubric is devoted to "sensations." The same feature should be found in a good Repertory.

2. This case also demonstrates the fallacy of the doctrine that in very severe pain narcotics and anæsthetics must sometimes be administered. *Similia similibus curantur* is not a mere rule of practice, not even only "the method of Hahnemann," but a *law of nature*, and therefore infallible. *Hamanum est errare*, and we are unfortunately not infallible in applying it; but should any one feel it necessary to resort to allopathic palliations, he should be honest, and lay the blame on his own want of knowledge, and not accuse Homœopathy of imperfections, nor falsely slander those who hasten to the defense of their beloved science and art.

3. The case also verifies Hahnemann's latest teaching that in many cases a repetition of the dose is essential. Improvement did not commence till after the second dose, nor was it marked till after the third. Had I only given one dose, and after waiting fifteen minutes concluded that Homœopathy had failed, though the simillimum had been given, and resorted to Chloroform, I should have committed a fatal error; and the error would have been nearly as great if I had concluded that the potency was too high and resorted to a lower one.

4. The error of the pathological school is also illustrated. This school maintains that a pathological similarity is the *ne plus ultra* of scientific therapeutics, and that we should only resort to semiological indications as a *dernier resort*, when our pathological knowledge is incomplete. Hence they regard the objective symptoms as of more importance than the subjective. But their arguments fail in this case. *Coccus* has not yet produced the pathological condition called gonorrhœa; and yet it cured, because it corresponded closely to the subjective symptoms. Very likely it has the power of producing gonorrhœa; but when we find that discharge recorded in provings shall we be able to prescribe any more efficaciously, or will that alone distinguish it from other remedies producing the discharge?

5. The great difference between Homœopathy and isopathy is that the former individualizes, while the latter generalizes. Under the principle of isopathy I should have given *Medorrhinum*, even though its voluminous provings do not contain the characteristic symptoms of the case. *Medorrhinum* has cured gonorrhœa *when the subjective symptoms of the patient corresponded with those of the provings*, but in other cases it has failed.

It has been argued by an able, though somewhat hasty and eccentric convert from allopathy, that "before any one could select *Syphilinum* with safety and with certainty, he must be first able to recognize syphilis in all its forms, and be able to differentiate between it and psora." In other words, *Syphilinum* will cure uncomplicated syphilis, but when complicated other (antipsoric) remedies will be necessary. But, at the risk of offending the writer, who, I know, does not like to be contradicted or to have his *dictum* called in question, I maintain that to diagnose complicated from uncomplicated syphilis is often an impossibility. In fact, I very much doubt whether, in the present diseased condition of the human race, any case be absolutely uncomplicated. The only scientific way to prescribe the nosodes is to prove them on healthy persons and to administer them to the sick according to such provings. If a nosode is indicated by the similarity of symptoms it will cure (or if a cure be impossible it will relieve), whether the patient be suffering from the "disease" from which the nosodes were taken or not; whether the "disease" be complicated or uncomplicated.

Always the *Law: Similia similibus curantur.*

